# Innovative strategies for prediction and targeted prevention of glaucoma in healthy vasospastic individuals: context of neurodegenerative pathologies

**DOI:** 10.1186/1878-5085-5-S1-A99

**Published:** 2014-02-11

**Authors:** Kristina Yeghiazaryan, Josef Flammer, Olga Golubnitschaja

**Affiliations:** 1 Department of Radiology, Rheinische Friedrich-Wilhelms-University of Bonn, Germany; 2 Department of Ophthalmology, University Hospital Basel, Switzerland

## Scientific background and objectives

Worldwide, 67 million patients are affected by the neurodegenerative eye disease glaucoma. Glaucomatous optic neuropathy (GON) is the second leading cause of permanent vision loss. GON is a chronic degenerative process the onset of which is not possible to monitor by currently existing diagnostic tools. Early treatment has been reported to be highly beneficial for well-timed treatment measures to slow-down the disease progression [1]. As reviewed by the authors, molecular pathomechanisms of glaucoma demonstrate both a considerable overlap and remarkable particularities to some other neurodegenerative disorders such as Alzheimer’s and Parkinson’s diseases [2]. Hence, *versus* controls the neuronal thread protein (NTP) demonstrates enhanced expression levels in glaucoma, patients with Down Syndrome, Alzheimer’s and some other neurodegenerative diseases indicating the axonal lesions. However, whereas the accumulation of *TAU*-protein is characteristic for Alzheimer’s disease and other tauopathies, glaucoma patients do not demonstrate an increase in the target protein *versus* controls [2]. A monitoring of the pathology-specific molecular patterns is particularly valuable to develop reliable diagnostic approaches before the manifest pathology. Predictive tests can specify individual predisposition for well-timed preventive measures.

## Results interpretation

**Figure 1:**
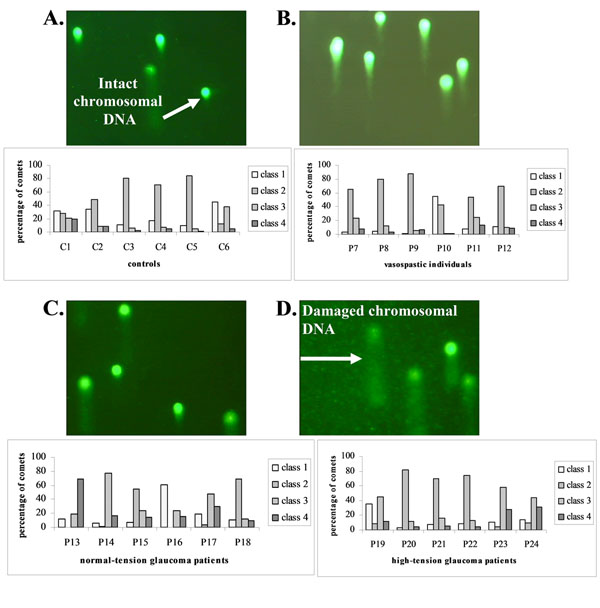
*Ex vivo* “Comet Assay“-analysis of DNA damage in circulating leukocytes of glaucoma patients and non-glaucomatous vasospastic individuals *versus* healthy controls [3]. Figures A-D give examples of images typical for A. control group, B. group of vasospastic individuals, C. group of normal-tension glaucoma patients, and D. group of high-tension glaucoma patients. Comet-patterns typical for healthy controls (A) show that chromosomal DNA is localised mainly to heads of comets (intact DNA). In contrast, images B, C, and D demonstrate clearly damaged DNA (visible comet-tails and diffuse comet-heads).

Comparative “Comet Assay” analysis revealed patterns of comets typical for glaucoma patients and some pattern similarities with vasospastic individuals, in contrast to controls as shown in Figure 1[3]. Although DNA damage in the vasospastic non-glaucomatous group is not found to be significantly increased *versus* healthy controls, DNA from vasospastic individuals showed highly group-specific comet-patterns with the degree of damage intermediate between healthy controls and glaucoma patients.

## Conclusions and outlook

These findings indicate “comet assay” profiling of DNA-damage in CL as a potentially powerful tool for the non-invasive early/predictive molecular diagnostics of glaucoma disease in vasospastic individuals. Furthermore, unrepaired DNA-damage in vasospastic individuals can lead to several pathologies different from glaucomatous optic nerve degeneration. This predisposition should be thoroughly investigated and the specificity of “Comet Assay”-patterns of vasospastic individuals should be validated comparing with patterns of other degenerative and non-degenerative pathologies. Thus, “Comet Assay”-analysis as a suitable tool for biomarkers has also been suggested for another neurodegenerative disorder – Alzheimer’s disease [4]. “Comet Assay”-analysis reveals enhanced DNA damage in both high- and normal-tension glaucoma [3]. Whether the level of DNA-damage correlates with disease severity, or not remains currently unclear. Further studies should also evaluate, whether a significant increase in DNA damage of leukocytes of glaucoma patients is caused by either disease specific stress factors, such as local ischemic/reperfusion events, and/or decreased capacity of DNA-repair machinery. There is some evidence for both eventualities: simultaneous up-regulation of *p53* (stress regulated gene) and down-regulation of *XPGC* (essential member of DNA-repair machinery) have been demonstrated *ex vivo* in CL of glaucoma patients [5] and represent potential molecular blood markers for the disease.

## Recommendations

A potential predisposition of vasospastic individuals to related pathologies should be thoroughly examined. This examination requires innovative strategies to cover following aspects:

• identification of possible similarities as well as dissimilarities in molecular pathways between healthy vasospastic individuals and potential related pathologies developed later in life

• specificity for predictive diagnostics of glaucoma pathology in predisposed vasospastic individuals should be strictly validated against several control groups including other neuro/degenerative diseases

• selection of molecular targets should be performed for vasospastic individuals in favour of non-invasive (blood test) diagnostic approaches followed by personalised treatment towards individual predisposition to single pathologies.

## References

[B1] Golubnitschaja O Yeghiazaryan K Flammer J Key molecular pathways affected by glaucoma pathology: Is predictive diagnosis possible? EPMA J 2010 1 237 244 10.1007/s13167-010-0031-4 23199062 PMC3405318

[B2] Golubnitschaja O Yeghiazaryan K Flammer J Mandel S Glaucomatous optic neuropathy: Risk assessment and potential targets for effective prevention and treatments tailored to the patient Neurodegenerative Diseases: Integrative PPPM Approach as the Medicine of the Future 2013 Springer Book series: *Advances in PPPM*

[B3] Moenkemann H Flammer J Wunderlich K Breipohl W Schild HH Golubnitschaja O Increased DNA breaks and up-regulation of both G(1) and G(2) checkpoint genes p21(WAF1/CIP1) and 14-3-3 sigma in circulating leukocytes of glaucoma patients and vasospastic individuals Amino Acids 2005 28 199 205 10.1007/s00726-005-0169-x 15723242

[B4] Migliore L Fontana I Colognato R Coppede F Siciliano G Murri L Searching for the role and the most suitable biomarkers of oxidative stress in Alzheimer's disease and in other neurodegenerative diseases Neurobiol Aging 2005 26 587 595 10.1016/j.neurobiolaging.2004.10.002 15708433

[B5] Golubnitschaja-Labudova O Liu R Decker C Zhu P Haefliger IO Flammer J Altered gene expression in lymphocytes of patients with normal-tension glaucoma Curr Eye Res 2000 21 867 876 10.1076/ceyr.21.5.867.5534 11262608

